# Validation of the Korean Stroop Test in Diagnosis of Minimal Hepatic Encephalopathy

**DOI:** 10.1038/s41598-019-44503-w

**Published:** 2019-05-29

**Authors:** Eileen L. Yoon, Dae Won Jun, Jae Yoon Jeong, Tae Yeob Kim, Do Seon Song, Sang Bong Ahn, Hee Yeon Kim, Young Kul Jung, Myeong Jun Song, Sung Eun Kim, Hyoung Su Kim, Soung Won Jeong, Sang Gyune Kim, Tae Hee Lee, Yong Kyun Cho, Jae-kwan Kim, Hokyoung Ryu

**Affiliations:** 1 Department of Internal Medicine, Sanggye Paik Hospital, Inje University College of Medicine, Seoul, 01757 Republic of Korea; 2 Department of Internal Medicine, Hanyang University Hospital, Hanyang University College of Medicine, Seoul, 04763 Republic of Korea; 3 Department of Internal Medicine, Hanyang University Guri Hospital, Hanyang University College of Medicine, Guri-si, 11923 Republic of Korea; 4 Department of Internal Medicine, New Hope Internal Medicine Clinic, Seoul, 03113 Republic of Korea; 5 Department of Internal Medicine, St. Vincent’s Hospital, College of Medicine, The Catholic University of Korea, Suwon, 16247 Republic of Korea; 6 Department of Internal Medicine, Nowon Eulji Medical Center, Eulji University College of Medicine, Seoul, 01830 Republic of Korea; 7 Department of Internal Medicine, Uijeongbu St. Mary’s Hospital, College of Medicine, The Catholic University of Korea, Uijeongbu-si, 11765 Republic of Korea; 8 Department of Internal Medicine, Korea University Medical Center, Ansan-si, 02841 Republic of Korea; 9 Department of Internal Medicine, Daejeon St. Mary’s Hospital, College of Medicine, The Catholic University of Korea, Daejeon, 34943 Republic of Korea; 10 Department of Internal Medicine, Hallym University Sacred Heart Hospital, Hallym University College of Medicine, Anyang-si, 14068 Republic of Korea; 11 Department of Internal Medicine, Kangdong Sacred Heart Hospital, Hallym University College of Medicine, Seoul, 05355 Republic of Korea; 12 Department of Internal Medicine, Soonchunhyang University College of Medicine, Soonchunhyang University Seoul Hospital, Seoul, 04401 Republic of Korea; 13 Department of Internal Medicine, Soonchunhyang University College of Medicine, Soonchunhyang University Bucheon Hospital, Bucheon-si, 14584 Republic of Korea; 14 Department of Internal Medicine, Konyang University College of Medicine, Daejeon, 35365 Republic of Korea; 15 Department of Internal Medicine, Kangbuk Samsung Hospital, Sungkyunkwan University School of Medicine, Seoul, 03181 Republic of Korea; 16 Department of Arts & Technology, Hanyang University, Seoul, 04763 Republic of Korea

**Keywords:** Hepatic encephalopathy, Laboratory techniques and procedures

## Abstract

The burden of minimal hepatic encephalopathy (MHE) is significant, but no universal criteria for diagnosis have been established. We aimed to validate the Korean Stroop Test for MHE screening. Chronic hepatitis B-related liver cirrhosis patients were recruited prospectively from 13 centers. The Korean Stroop Test consisted of two Stroop-off states (color and word) and two Stroop-on states (inhibition and switching). Accuracy adjusted psychomotor speed (rate correct score) of these tests were analyzed. Sex- and age- adjusted rate correct scores of these tests were rated as the Korean Stroop Score (K-Stroop score). MHE was diagnosed when Portosystemic Encephalopathy Syndrome Test (PHES) scores were below −4. A total of 220 liver cirrhosis patients and 376 healthy controls were enrolled. Prevalence of MHE was 20.6% in cirrhosis patients. Rate correct scores and the K-Stroop score showed significant differences between healthy controls, cirrhosis patients without MHE, and cirrhosis patients with MHE. The rate correct score of the K-Stroop score was 0.74 (95% Confidence Interval: 0.66–0.83, *P* < 0.001). Female gender and the K-Stroop score were significant for MHE diagnosis. The Korean Stroop Test is simple and valid for screening of MHE.

## Introduction

Hepatic encephalopathy (HE) is defined as a brain dysfunction caused by liver insufficiency and/or portosystemic shunt^1^. Minimal HE (MHE) lies along the spectrum of neurocognitive impairment in liver cirrhosis (LC)^1^. Patients with MHE are asymptomatic, but they exhibit abnormal findings in neuropsychological and/or neurophysiological tests by definition^2^. According to previous studies, the prevalence of MHE has been reported to vary widely from 30–85% and is significantly associated with liver function of the population and the diagnostic tests used^1^. However, it is evident that at least one-third of cirrhotic patients exhibit MHE in Korea^3,4^. Patients with MHE have been reported to have a poor prognosis in that they experience poor quality of life and have a higher risk of traffic violations and accidents^5,6^. Furthermore, a MHE episode can predict the development of overt HE, death and hospitalizations^7^. Therefore, it is important to test for MHE in patients who are at risk, even though they have no overt symptoms or signs of cognitive dysfunction^1^.

There are many tests for MHE, which can be largely categorized as paper-pencil-based tests, computerized tests, and neurophysiological tests^2^. However, no universal criteria for diagnosis have yet been established. The Portosystemic Encephalopathy Syndrome Test (PHES) is the most validated test worldwide and is considered the gold standard for the diagnosis of MHE^8,9^. However, paper-pencil tests are burdensome to use as screening tests in asymptomatic patients in real-life practice. Ideal screening tests for MHE should be simple to use, have an objective outcome, be less time-consuming, be mobile-based, independent of specialists’ interpretation, and free from copyrights and fees. Additionally, they should have local normal values, and validated data. The Stroop task has been analyzed in several studies of MHE^10–12^. Recently, a smartphone-based Stroop test, the Encephalapp, has been developed and validated for the screening and diagnosis of covert HE in the United States^13–16^. Although the Encephalapp is free of charge, differences in language make it difficult to use the Encephalapp outside the United States. Furthermore, we need the normative data and validating process in the Korean LC population to apply the Encephalapp in clinical practice. Therefore, we developed and aimed to validate the K-Stroop Test for the screening and diagnosis of MHE in Korea.

## Results

### Baseline characteristics

A total of 376 healthy controls were enrolled in this study. Approximately half of the control group was male (n = 190). The mean age was 43 years and the mean years of education was 14 years. Healthy controls were evenly distributed in each subgroup divided by sex and 10-year intervals of age (Supplementary Table S1).

A total of 220 LC patients were enrolled. Sixty-seven percent of the patients were male (n = 148) (Table 1). The mean age of the LC patients was 54 years and the years of education were 11 years. Thus, there were significant differences from healthy controls (both *P* < 0.001). The model for end-stage liver disease (MELD) score of LC patients was 9 ± 3. The prevalence of MHE based on conventional PHES in LC patients was 20.5%. The mean years of education was shorter in LC patients with MHE than in LC patients without MHE. Serum albumin levels were lower in LC patients with MHE. However, sex, age, platelet counts, prothrombin time, ALT, total bilirubin, sodium, ammonia, and MELD were were not different between the two groups.

**Table 1:** Baseline characteristics of healthy controls and liver cirrhosis patients.

	Healthy controls (n = 376)	LC without MHE (n = 175)	LC with MHE (n = 46)
Male^*^	190 (50.6%)	123 (70.3%)^†^	25 (55.6%)
Age (years)	43 ± 14	53 ± 7^†^	55 ± 7^‡^
Education (years)	14 ± 3	12 ± 3^†^	10 ± 3^‡§^
Platelets (×10^3^/uL)	NA	123 ± 56	111 ± 53
Prothrombin time (INR)	NA	1.18 ± 0.22	1.21 ± 0.20
Albumin (g/L)	NA	4.2 ± 0.5	4.0 ± 0.6^§^
ALT (IU/L)	NA	30 ± 17	31 ± 29
Total bilirubin (mg/dL)	NA	1.2 ± 0.8	1.4 ± 1.2
Sodium (mEq/L)	NA	140 ± 3	139 ± 4
Ammonia (mmol/L)	NA	100 ± 61	88 ± 42
MELD	NA	9 ± 3	10 ± 3

Numbers are shown as mean ± standard deviation.

^*^Percentage of number out of each group is shown in parentheses.

^†^*P* value < 0.05 in comparison of values between HCs vs. LC without MHE.

^‡^*P* value < 0.05 in comparison of values between HCs vs. LC with MHE.

^§^*P* value < 0.05 for comparison of values between LC without MHE vs. LC without MHE.

ALT; alanine aminotransferase; LC: liver cirrhosis; MELD: model for end-stage liver disease; MHE: minimal hepatic encephalopathy; NA: not available.

### Correlation of the K-Stroop Test results with demographic data in healthy controls

In healthy controls, age and education years showed significant negative correlation (*r* = −0.43, *P* value < 0.001). The rate correct scores (RCS) of each test were calculated by the formula described in the methods section (Table 2). RCS - Color Off (RCS-C), RCS – Word Off (RCS-W), RCS – Inhibition On (RCS-I), and RCS – Switching On (RCS-Sw) showed negative correlations with age (all *P* < 0.001) and positive correlation with education years (all *P* < 0.001) in healthy controls. The K-Stroop score showed nonsignificant or very weak correlation with age and education years. There was no significant difference related to gender on RCS values of these four tests or the K-Stroop score.

**Table 2:** Correlation of the K-Stroop Test results with demographic data in healthy controls.

	Age		Education	
	*r*	*P* value	*r*	*P* value
Age (years)	1			
Education (years)	−0.43	<0.001	1	
RCS				
Color Off	−0.69	<0.001	0.47	<0.001
Word Off	−0.69	<0.001	0.41	<0.001
Inhibition On	−0.70	<0.001	0.50	<0.001
Switching On	−0.66	<0.001	0.40	<0.001
K-stroop score	0.03	0.555	−0.13	0.012

*r* = Pearson correlation coefficient.

Statistical significance was set at a *P* value < 0.05.

RCS: rate correct score.

### Correlation of the K-Stroop Test results with clinical parameters in LC patients

In LC patients, the age and education years showed significant negative correlation (Table 3). As in healthy controls, the RCS of each test in LC patients showed significant negative correlation with age and weak positive correlation with education years. There was no significant gender effect on RCS values of these four tests or the K-Stroop score in LC patients. The RCS of each test showed positive correlation with PHES score. The K-Stroop score did not show significant correlation with age or education years. However, it showed negative correlation with the PHES score. PHES score, the RCS and the K-Stroop score showed no significant correlation with MELD.

**Table 3:** Correlation of the K-Stroop results with clinical parameters in liver cirrhosis patients.

	Age		Education		MELD		PHES score	
	*r*	*P* value	*r*	*P* value	*r*	*P* value	*r*	*P* value
Age (years)	1							
Education (years)	−0.34	<0.001	1					
MELD	−0.03	0.651	0.06	0.456	1			
PHES score	−0.19	0.004	0.16	0.018	−0.15	0.038	1	
RCS								
Color Off	−0.42	<0.001	0.38	<0.001	−0.14	0.061	0.38	<0.001
Word Off	−0.35	<0.001	0.27	<0.001	−0.16	0.032	0.54	<0.001
Inhibition On	−0.37	<0.001	0.37	<0.001	−0.08	0.299	0.50	<0.001
Switching On	−0.37	<0.001	0.30	<0.001	−0.08	0.280	0.34	<0.001
K-Stroop score	−0.02	0.750	−0.22	0.001	0.16	0.027	−0.40	<0.001

*r*: Pearson correlation coefficient; MELD: model for end-stage liver disease; PHES: portosystemic encephalopathy syndrome test; RCS: rate correct score.

Statistical significance was set at a *P*-value < 0.05.

### Comparison of the K-Stroop Test results between healthy controls and liver cirrhosis patients with or without MHE based on conventional PHES

RCS values of LC patients without MHE were significantly lower than those of healthy controls (all *P* < 0.001) (Table 4). Comparison between healthy controls and LC with MHE patients showed similar patterns (all *P* < 0.001). LC with MHE patients showed lower RCS values in each test when compared with those of LC without MHE patients.

**Table 4:** Comparison of the RCS of each test and K-Stroop score among healthy controls, liver cirrhosis patients without MHE, and liver cirrhosis patients with MHE based on conventional PHES.

	HCs (n = 376)	LC without MHE (n = 175)	LC with MHE (n = 46)
RCS			
Color Off	3.7 ± 0.8	2.9 ± 0.8	2.3 ± 1.1
Word Off	4.1 ± 0.7	3.7 ± 0.6	3.0 ± 1.0
Inhibition On	3.4 ± 0.9	2.7 ± 0.9	1.7 ± 1.0
Switching On	2.7 ± 0.9	1.8 ± 0.8	1.3 ± 0.7
K-Stroop score	0.3 ± 0.7	0.8 ± 1.0	1.4 ± 0.2

All the *P* values were less than 0.001 in comparison of values between any two groups (i.e. HCs vs. LC without MHE, HCs vs. LC with MHE, and LC without MHE vs. LC with MHE).

HCs: healthy controls; LC: liver cirrhosis; MHE: minimal hepatic encephalopathy; RCS: rate correct score.

Mean values of the K-Stroop score were significantly different when compared between healthy controls, LC patients without MHE, and LC patients with MHE (all *P* < 0.001).

### Receiver operating curves of the K-Stroop results for diagnosis of MHE in liver cirrhosis patients

Four types of RCS in each test produced an area under the curve (AUC) of 0.68–0.80 (all *P* < 0.001) (Table 5). The K-Stroop score produced an AUC of 0.74 (95% Confidence Interval (C.I.) 0.66–0.83, *P* < 0.001). AUC for RCS-I showed the highest level at 0.80 (95% C.I. 0.73–0.88, *P* < 0.001). The cut-off point for the highest Youden’s index value was 2.08 for RCS-I with 80.0% sensitivity and 73.1% specificity. The cut-off point for the highest Youden’s index value for diagnosis of MHE in the K-Stroop score was 0.5 with 82.6% sensitivity and 53.1% specificity. A K-Stroop score of 1.5 showed 52.2% sensitivity and 79.7% specificity.

**Table 5:** Receiver operating curves of the K-Stroop Test results for diagnosis of MHE in LC patients.

	AUC	*P* value	95% C.I.
RCS			
Color Off	0.68	<0.001	0.59–0.77
Word Off	0.78	<0.001	0.71–0.86
Inhibition On	0.80	<0.001	0.73–0.88
Switching On	0.70	<0.001	0.61–0.79
K-Stroop score	0.74	<0.001	0.66–0.83

AUC: area under curves; C.I: Confidence interval; RCS: rate correct score.

Statistical significance was set at a *P* value < 0.05.

### Predictive factors for the presence of MHE in LC patients

Sex, age, years of education, RCS-I, K-Stroop score, and MELD were entered into logistic regression analysis. Female sex, years of education, RCS-I, and K-Stroop score were significant variables in univariate analysis as predictive factors of MHE in LC patients (Table 6). Female sex was the significant variable in multivariate analysis with the range of odds ratio between 2.79–2.91. Both the RCS-I and K-Stroop scores were significant predictive factors for the presence of MHE in LC patients independent of the MELD.

**Table 6:** Logistic regression analysis for predictive factor of minimal hepatic encephalopathy in liver cirrhosis patients.

	Univariate		Multivariate I		Multivariate II	
	Odds ratio (95% C.I.)	*P* value	Odds ratio (95% C.I.)	*P* value	Odds ratio (95% C.I.)	*P* value
Female sex	1.99 (1.02–3.86)	0.043	2.91 (1.02–4.73)	0.045	2.79 (1.26–6.16)	0.011
Age (years)	1.05 (1.00–1.10)	0.069	0.98 (0.92–1.04)	0.456	1.05 (0.99–1.11)	0.123
Education (years)	0.89 (0.80–0.99)	0.030	1.01 (0.89–1.14)	0.936	1.01 (0.89–1.15)	0.885
RCS–Inhibition On	0.33 (0.22–0.50)	<0.001	0.31 (0.20–0.49)	<0.001		
K-stroop score	2.06 (1.56–2.71)	<0.001			2.29 (1.67–3.15)	<0.010
MELD	1.08 (0.99–1.21)	0.178				

C.I.: confidence interval. MELD: model for end-stage liver disease; RCS: rate correct score.

Statistical significance was set at a *P* value < 0.05.

### Analysis for test-retest reliability and comparison of devices (smartphone vs. tablet)

Four types of RCS for each test were analyzed in 63 healthy controls who repeated the K-Stroop Test after a short period to assess test-retest reliability. Test results were compared between visit 1 and visit 2 for each device, i.e. smartphone and tablet, and showed no differences overall. In addition, the K-Stroop Test results were compared between smartphone and tablet devices at each visit, i.e. visit 1 and visit 2, and the results also showed no significant differences.

To assess learning effects, we compared the RCS during the familiarization trial, 1^st^ test, and 2^nd^ test at visit 1, and observed differences within subject as repetitions progressed for each test (all *P* < 0.001). The RCS-Sw showed significant differences between the values of the 1^st^ and 2^nd^ tests (Table 7).

**Table 7:** Analysis for learning effects and comparison of the K-Stroop Test results when repeating tests.

	Trial	1^st^ Test	2^nd^ Test	*P* value^*^	*P* value^†^	*P* value^‡^
RCS						
Color Off	4.0 ± 0.7	4.2 ± 0.7	4.3 ± 0.7	<0.001	0.006	0.021
Word Off	4.3 ± 0.7	4.3 ± 0.7	4.5 ± 0.6	<0.001	0.663	<0.001
Inhibition On	3.7 ± 0.9	4.0 ± 0.7	4.0 ± 0.9	<0.001	<0.001	0.293
Switching On	3.0 ± 0.8	3.2 ± 1.1	3.4 ± 0.9	<0.001	<0.001	<0.001

^*^P value for comparison of the values of three repetitive measurements, i.e. values of trial, 1^st^ test, and 2^nd^ test, in same respondent analyzed by repetitive measurement analysis of variance (ANOVA).

^†^*P* value for comparison of the values of two repetitive measurements of the trial and 1^st^ test in the same respondent analyzed by paired sample t- test.

^‡^*P* value for comparison of the values of two repetitive measurements of the 1^st^ test and 2^nd^ test in the same respondent analyzed by paired sample t- test.

Statistical significance was set at a *P* value < 0.05.

RCS: rate correct score.

## Discussion

Epidemiologic studies regarding MHE in Korea are limited due to the lack of normalized and validated data for appropriate diagnostic tools. Seo *et al*. reported that the prevalence of MHE in LC patients was 25.6%, of which 20.2% were Child-Pugh class A, 42.9% Child-Pugh class B, and 60.0% Child-Pugh class C based on the Korean version of the conventional PHES^4^. The authors provided normative data of the Korean version of the conventional PHES based on 200 healthy Korean subjects^4^. However, this version of the PHES has an obstacle for its widespread use in real-life practice, as approval from the copyright holder of conventional PHES should be obtained before use. Jeong *et al*. adopted and modified the conventional version of PHES into a new ‘copyleft’ paper-pencil test in 2017^3^. They established normative reference data based on 315 healthy subjects and validated the results in a small group of cirrhotic patients^3^. The prevalence of MHE was estimated to be 37.5% based on this new Korean paper-pencil test^3^. Although MHE is asymptomatic, it has a sizeable prevalence and poor prognostic implications in LC patients. Nevertheless, there is no consensus on which test should be used to diagnose MHE in real-life practice. Furthermore, recent guidelines suggest that either the computerized test or neurophysiological testing should be used alongside the paper-pencil test (i.e., the PHES) for multicenter studies. However, it is difficult to make good use of neurophysiological tests, such as electroencephalography, critical flicker frequency, and evoked potentials, as they are expensive, time-consuming, and dependent on the specialist’s interpretation. Therefore, alternative computerized tests are required to carry out multicenter studies of MHE in Korea.

In this context, the EncephalApp is a computerized test, which has been validated for the diagnosis of MHE in the United States. It is based on the Stroop effect universally present in individuals able to read letters. Theoretically, an increase in the latency and the number of errors in response to incongruent conditions, relies on a higher strength of reading response than a naming response^17^. Nevertheless, there are some obstacles to the use of the original EncephalApp as a diagnostic tool of MHE in Korea, as it lacks Korean normative reference and validated data. The K-Stroop Test has been modified from the EncephalApp and has been developed to meet the needs of both real-life practice and multi-center studies as it is computer-based, highly accessible by web, less time-consuming, and free from copyright issues.

In the EncephalApp, On Time + Off Time, rather than accuracy of the results, was the best element to discriminate MHE patients among LC patients with an AUC of 0.91^14,15^. Based on these results, clinicians could be confused as to whether the Stroop task should be used for the diagnosis of MHE when the times for response were more significant than numbers of errors irrespective of whether Stroop states are “Off” or “On”. We assume this confusion may be related to the characteristics of the original EncephalApp. The EncephalApp assesses accuracy by repeating tasks until the subject correctly answers ten problems in a row. Emphasis is given to the time taken to repeat the test after the subject has made a mistake, giving less opportunity for accuracy. As a result, the patient solves at least 100 problems to accomplish the EncephalApp even if no mistakes are made. We compared the RCS of each test, assessing reaction times weighted by accuracy. As a result, the K-Stroop Test required only 40 problems to differentiate patients with MHE.

The RCS of each test were shown to be significantly correlated with either age or years of education in both healthy controls and LC patients. The K-Stroop scores are the number of tests where the result lies more than 1.5 standard deviation (SD) below the mean value of each subgroup, divided according to sex and 10-year intervals of age (range 0 to 4). Unlike the EncephalApp, which proposed time-fixed cut-off criteria regardless of sex and age, we analyzed the data based on the mean and SD values collected from Korean healthy controls. We could not adjust for years of education, but we assume that it could be adjusted by the fact that the K-Stroop score showed very weak correlation with the years of education (r = −0.22, *P* value 0.001). This could be related to the tendency to a lower level of education in older subjects and vice versa.

Interestingly, both PHES and K-Stroop scores showed a very weak correlation with MELD (*r* values of −0.15 for the PHES score and 0.16 for the K-Stroop score) differing from results based on the higher prevalence rate in Child Pugh C than Child Pugh A. Perhaps the prevalence of MHE could have been underestimated because clinicians do not consider the possibility of MHE in the asymptomatic population with good liver function.

There were significant negative correlations between the K-Stroop and PHES scores, but agreement between the tests was poor, based on the cut-off point of 1.5 (highest specificity 79.7%) for K-Stroop score (kappa 0.29, *P* < 0.001). This may be because HE is associated with multidimensional dysfunction and PHES only measures two (psychomotor speed and visuospatial demand) of seven domains in cognitive function (psychomotor speed, working memory, verbal memory, visuospatial ability, visual memory, language, reaction time, and motor function)^18–21^. Furthermore, PHES itself does have false positive or false negative rates even though it is the conventional gold standard in the diagnosis of MHE^22^. Therefore, it is possible for a subgroup of patients to show significantly poor psychometric performance compared to healthy controls by the Stroop test but not the PHES test. The clinical significance of Stroop abnormality requires long term follow up.

Although the AUCs of RCS-W or RCS-I were similar, we speculate that the Inhibition On test is equivalent to a Stroop test and would thus have better discriminating power than the Word Off test alone. Both the RCS-I and K-Stroop scores were significant predictive factors of MHE in LC patients regardless of liver function in multivariate analysis in our study. Therefore, we suggest that RCS-I can be used for rapid screening of a patient with a cut off of 2.08 regardless of sex and age. In patients with high suspicion of MHE, results can be judged by the K-Stroop score adjusted for sex and age. The cut-off of 1.5 can be used for the diagnosis of MHE given the high specificity. Unlike previous studies where gender susceptibility for MHE was not significant, female gender was another predictive factor for MHE in our study. This needs to be further evaluated.

There was good test-retest reliability in the K-Stroop Test. A learning effect was found based on the RCS for each test in healthy controls. Thus, a familiarization process is required for respondents to understand and practice the tests. The type of device used, whether smartphone or tablet, had no effect on the outcomes of the K-Stroop Test.

This is the first trial assessing the Stroop Test for the diagnosis of MHE in LC patients of a non U.S population. Additionally, this is the first version of the Stroop test to be validated in LC patients with or without MHE in Korea. The normative data of the K-Stroop Test is essential to diagnose MHE in a real-life setting and to carry out multicenter studies. However, there were several limitations to this study. First, the definition of healthy controls was not strict. We relied upon the subjective answers of healthy controls who did not meet the exclusion criteria. Laboratory findings were not available in healthy controls. Second, we compared the result of the K-Stroop Test to the PHES but the PHES is the provisional gold standard test. Eventually, these tests should be helpful not only in the diagnosis of MHE, but also in the formulating of prognosis. Therefore, a longitudinal study comparing prognoses of patients who were diagnosed MHE by only the PHES, only the K-Stroop Test, and both the PHES and the K-Stroop Test are required. Third, the K-Stroop Test does not present the opportunity in trials for respondents to practice and understand each step of the test before being assessed. This can lower the power of discrimination especially in the process of the Switching On test. Fourth, the position of each example, e.g. red, yellow, green, and blue, was fixed for each test question. We recently updated the K-Stroop Test (accessible via http://encephalopathy.or.kr), to improve these limitations and are planning to set normative data for the updated version of the K-Stroop Test in the near future. In conclusion, the mobile-based K-Stroop Test is a simple, handy, objective, and valid method to screen and diagnose for MHE in real-life practice. The K-Stroop Test may serve as a diagnostic tool for MHE assessment in multi-center studies alongside paper-pencil tests in Korea.

## Methods

### Study design

Chronic hepatitis B (CHB)-related LC patients were prospectively recruited from 13 academic hospitals from January 2016 to December 2017. All participants completed the conventional PHES and the K-Stroop Test. PHES was tested with paper and pencil and consisted of a number connection test (NCT)-A, NCT-B, digit symbol test, serial dotting test, and line tracing test (time and error) (Supplementary Table S2). MHE was diagnosed when PHES scored below −4. The normative data of PHES in the Korean population was adopted from a recent Korean study^4^.

### Study endpoints

The primary endpoint was to validate the K-Stroop Test for screening of MHE in LC patients. Other endpoints were to create normative data for the K-Stroop Test in Korea and to study reliability and inter-device correlation in healthy controls.

### Liver cirrhosis patients

LC was diagnosed by either of the following criteria: (1) biopsy-proven LC or (2) clinical diagnosis such as presence of surface nodularity of liver, splenomegaly, and portosystemic shunt on abdominal imaging, esophageal or gastric varices in endoscopy, thrombocytopenia (platelet counts <100,000/mm^3^), hypoalbuminemia (albumin ≤3.5 g/dL), and prothrombin time prolongation (international normalized ratio [INR] ≥1.3) in laboratory findings. Inclusion criteria were (1) age between 19–65 years, (2) CHB-related LC. Patients were excluded from the study if they met the following criteria: (1) age >65 years old, (2) previous history of overt HE (West Haven grade >2), (3) alcohol intake of >210 g per week for men and 140 g per week for women in the 2 years prior to the study, (4) taking neurologic or psychological medications, (5) history of systemic infection within 6 weeks, (6) history of gastrointestinal bleeding within 6 weeks, (7) presence of viable hepatocellular carcinoma or other malignant disease, or (8) color blindness.

### Healthy controls

We prospectively recruited voluntary healthy controls to establish normative data for the K-Stroop Test over the same period from a single center. Adults aged 19 to 65 years were included. Exclusion criteria were: (1) uncontrolled chronic disease within 6 months, (2) suspicious symptoms or previous history of dementia, (3) taking neurologic or psychological medications, (4) alcohol intake more than 210 g per week for men, and 140 g per week for women in recent two years, and (5) color blindness. Healthy controls also completed the K-Stroop Test after obtaining informed consent. Subgroups were divided into 10 groups according to sex and 10-year intervals of age (20–29, 30–39, 40–49, 50–59, 60–69).

### Design of the Korean Stroop Test

Each test presented 10 questions and measured time to finish each test and the rate of correct response during each test (accessible via http://166.104.16.213). The Stroop-off states consisted of a “Color Off” test and “Word Off” test, which required a reading response to congruent stimuli without provoking a Stroop interference effect (Supplementary Fig. S3). The “Color Off” test presented colored symbols “####” and gave instructions to choose the color of the symbols. The “Word Off” test presented color words in Korean in black-colored lettering and gave instructions to choose the name of the color. Stroop “On” states consisted of an “Inhibition On” test and “Switching On” test, which required naming responses to incongruent stimuli to provoke Stroop interference. The “Inhibition On” test presented a color word in a mismatched color and gave instructions to choose the color of the word (e.g. the answer is “green” to “red” in a green color). The “Switching On” test alternatively presented an Inhibition On test or Switching test. The Switching On test presented a color word with either matched or mismatched color in a box, and gave instruction to choose the color word itself irrespective of the color of the word (reading response). Four examples were in fixed spots, which were red, yellow, green, and blue. The K-Stroop Test was tested with an 8-inch tablet computer.

### Interpretation of the Korean Stroop Test

The Stroop test is a test of psychomotor speed and cognitive flexibility. The Stroop test measures Stroop effect which shows slower, error-prone responses to incongruent stimuli (e.g., color-word printed in a mismatched color)^17^. To combine the information of speed and accuracy provided by the test, we analyzed the RCS in Color Off test, Word Off test, Inhibition On test, and Switching on test^23^. RCS is calculated by the formula as below and it can be interpreted as the rate of correct response in one second^24^.

$${\rm{Rate}}\,{\rm{correct}}\,{\rm{score}}\,({\rm{RCS}})=\frac{{\rm{the}}\,{\rm{number}}\,{\rm{of}}\,{\rm{correct}}\,{\rm{responses}}}{{\rm{\Sigma }}\,response\,time}$$

The K-Stroop Test score was calculated as the number of tests with an impaired performance under −1.5 standard deviations (SDs) from the mean value based on the age groups (20–29, 30–39, 40–49, 50–59, and 60–69) of healthy controls in each four test (Supplementary Table S2).

### Test-retest reliability and comparison of devices: smartphone versus tablet

Among the healthy controls, 63 individuals voluntarily participated in the test-retest reliability protocol. They repeated the K-Stroop Test after 22 ± 16 days (range: 14–114 days). At each visit, they started with a familiarization exercise to exclude learning effect. Half of the individuals were first tested using a smartphone and second with a tablet on visits 1 and 2. The other half of the participants first started with the tablet and second with a smartphone to compare results across the devices.

### Biochemical analysis

Blood test samples of LC patients were taken on the day of the PHES performance. These included complete blood cells, prothrombin time, serum albumin, aspartate aminotransferase (AST), alanine aminotransferase (ALT), total bilirubin, creatinine and ammonia.

### Statistical analysis

Categorical and continuous variables are expressed as the mean ± standard deviation and number (%), respectively. These variables were analyzed using the Chi-square test or Fisher’s exact test and Student’s independent *t*-test. Pearson’s correlation test was used to assess the correlation between the results of the K-Stroop Test and demographic-/clinical- parameters, such as age, education, serum ammonia, MELD, and PHES score, etc. We compared receiver operating characteristic (ROC) curves of the K-Stroop results to assess the most accurate parameter of the MHE based on conventional PHES in LC patients. Significant variables by univariate analysis in logistic regression were entered into multivariate regression analysis. For the analysis of the learning effect and inter-device compatibility (i.e. smartphone and tablet), repetitive measurement ANOVA was used to compare results of the familiarization trial, the 1^st^ test, and 2^nd^ test. Statistical significance was set at a *P*-value < 0.05. Statistical analysis was performed using SPSS 21.0 software (SPSS, Inc., an IBM Company, Chicago, IL, USA).

### Ethics statement

This study was approved by the Institutional Review Board of all the participating centers, which are Sanggye Paik Inje University Hospital, Hanyang University Hospital, Hanyang University Guri Hospital, St. Vincent’s Hospital, Nowon Eulji Medical Center, Uijeongbu St. Mary’s Hospital, Korea University Medical Center, Daejeon St. Mary’s Hospital, Hallym University Sacred Heart Hospital, Kangdong Sacred Heart Hospital, Soonchunhyang University Seoul Hospital, Soonchunhyang University Bucheon Hospital, Konyang University Hospital, and Sungkyunkwan University Kangbuk Samsung Hospital. The research was performed in accordance with relevant guidelines and regulations. Written informed consent was obtained from all participants and/or their legal guardians who are enrolled in this study

## Supplementary information

Supplementary tables & a figure

## Data Availability

The datasets generated during and/or analyzed during the current study are available from the corresponding author on reasonable request.
