# Supplementary material for: Validation of the Korean Stroop Test in Diagnosis of Minimal Hepatic Encephalopathy
Source: Sci Rep. 2019 May 29;9:8027. doi: 10.1038/s41598-019-44503-w (PMC6541633; doi:10.1038/s41598-019-44503-w)
Supplement: Supplementary file 1 — Supplementary tables & a figure [file 41598_2019_44503_MOESM1_ESM.pdf]

**Title: Validation of the Korean Stroop Test in Diagnosis of Minimal Hepatic Encephalopathy**

Eileen L Yoon[1], Dae Won Jun[2], Jae Yoon Jeong[3], Tae Yeob Kim[4], Do Seon Song[5], Sang Bong Ahn[6], Hee Yeon Kim[7], Young Kul Jung[8], Myeong Jun Song[9], Sung Eun Kim[10], Hyoung Su Kim[11], Soung Won Jeong[12], Sang Gyune Kim[13], Tae Hee Lee[14], Yong Kyun Cho[15], Jae-kwan Kim[16] & Hokyoung Ryu[16]

**Supplementary Table S1.** Number of healthy controls enrolled in each group categorized by sex and age.

| | Male | Female | Total |
|---|---|---|---|
| 20–29 years | 43 | 37 | 80 |
| 30–39 years | 34 | 55 | 89 |
| 40–49 years | 43 | 31 | 74 |
| 50–59 years | 39 | 34 | 73 |
| 60–69 years | 31 | 29 | 60 |
| Total | 190 | 186 | 376 |

**Supplementary Table S2. Components and ratings for (a) the Portosystemic Encephalopathy Syndrome Test (PHES) scores and (b) the Korean stroop score**

**(a)**

| | Results | Score* | PHES score |
|---|---|---|---|
| Number connection test-A | a (seconds) | a’ | a’+b’+c’+d’+e’ |
| Number connection test –B | b (seconds) | b’ | |
| Digit symbol test | c (points) | c’ | |
| Serial dotting test | d (seconds) | d’ | |
| Line tracing test | e (seconds and error score) | e’ | |

**(b)**

| | Results† | RCS‡ | Score§ | Korean Stroop score |
|---|---|---|---|---|
| Color Off | a | a’ | a” | a”+b”+c”+d” |
| Word Off | b | b’ | b” | |
| Inhibition On | c | c’ | c” | |
| Switching On | d | d’ | d” | |

* Scores are rated based on the results of each tests and normative data of the Korean healthy control group. Results of each test within ± 1 standard deviations (SDs) from the mean was scored as 0 points. Results between +1 and +2 SDs, between +2 and

+3 SDs, and more than +3 SDs from the mean were scored -1, -2, and -3 points, respectively.

† Results are given as time (speed) in seconds and correct response rates.

‡ RCSs are calculated by the formula as follows.

$$\mathrm{RCS} = (\text{the number of correct responses})/(\Sigma\ \text{response time})$$

§ RCSs less than -1.5 SDs from the mean values of Korean healthy control group were scored 1 point. RCSs equal to or more than -1.5 SDs from the mean were scored 0 point.

RCS: rate correct score

**Supplementary Figure S3. Examples of the Korean Stroop (K-Stroop) Test**

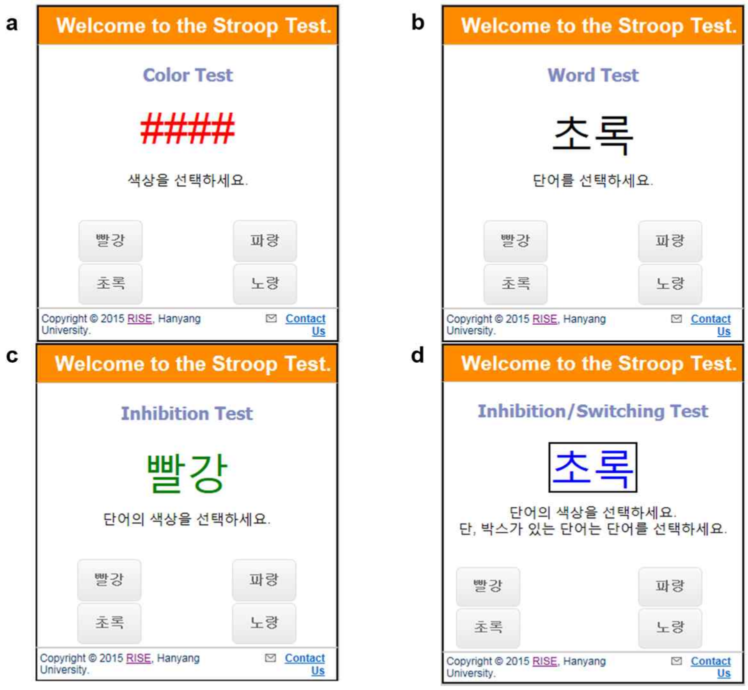


The K-Stroop Test consists of 4 tests: Color Off-, Word Off-, Inhibition On-, and Switching On- tests. The Color Off- and Word Off- tests are compatible with the Stroop-off state, while Inhibition On- and Switching On- tests are compatible with the Stroop-on state

a: The “Color Off” test presents colored symbols “####” and asks respondents to choose the color of the symbols.

b: The “Word Off” test presents color words in black font in Korean and asks respondents to choose the name of the color.

c: The “Inhibition On” test presents color words in a mismatched color font and asks respondents to choose the color of the word (e.g. the answer is “green” for “red” in a green font).

d: The “Switching On” test alternatively presents the Inhibition On test or switching test. The Switching On test presents the color word with either matched or mismatched color font in a box, and asks the respondent to choose the color word itself irrespective

of the color of the word.
